# Supplementary material for: Are carbon emissions trading and green financial instruments synergistic? -Comprehensive quantitative research based on content analysis
Source: PLoS One. 2024 Mar 7;19(3):e0298601. doi: 10.1371/journal.pone.0298601 (PMC10919601; doi:10.1371/journal.pone.0298601)
Supplement: S1 Data — (ZIP) [file pone.0298601.s001.zip › Data/地级市Z_F工作报告.zip!/║■▒▒/╥╦▓2╩╨/└┤╫╘╣1⁄2╓┌║┼í╛┬φ┐╦╩2╛▌═°í┐_2018.docx]

各位代表：

现在，我代表市人民政府向大会报告工作，请予审议，并请各位市政协委员和列席人员提出意见。

一、2017年工作回顾

刚刚过去的一年，是宜昌发展极为困难的一年，更是全市上下强力攻坚的一年。面对严峻复杂的经济形势，面对转型升级的艰巨任务，在市委坚强领导下，我们以习近平新时代中国特色社会主义思想为指导，坚定不移贯彻新发展理念，认真落实省第十一次党代会、市第六次党代会部署，坚持稳中求进工作总基调，化压力为动力，视难题为考题，抓重点、补短板、强弱项，务实重行，砥砺奋进，开创了“绿色、转型、跨越、小康”新局面。预计全市地区生产总值增长2%，规模以上工业增加值增长1%，社会消费品零售总额增长5%，固定资产投资2544亿元。

——产业转型升级迈出坚实步伐。痛下决心、壮士断腕，强力推进沿江134家化工企业“关、转、搬”，首批依法关停企业25家，传统化工占化工产值比重下降1.2个百分点，精细化工占比提高到20.3%。单位生产总值能耗下降7.14%、地耗下降5.32%、水耗下降13.7%。第三产业增加值占生产总值比重提高1个百分点。我市培育发展战略性新兴产业、推动特色优势产业转型升级成效明显，受到国务院通报表扬。

——三大攻坚战取得阶段性胜利。污染防治全面加强，按期完成中央环保督察交办问题整改41项，主要河流水质总体良好，PM10、PM2.5浓度分别下降9.3%、6.5%，环境空气质量优良天数达到258天，同比增加11天。精准脱贫扎实推进，实现81705人脱贫、60个村出列、18315人易地扶贫搬迁，预计远安县脱贫摘帽，光伏扶贫经验在全国推广。风险防范化解有力有效，宜昌轿车、联邦电缆等项目成功盘活，宜化集团改革解困稳步推进，依法处置“僵尸企业”22家，化解了一批经济和金融领域风险隐患。金融机构不良贷款率降至1.76%。

——供给侧结构性改革纵深推进。压减磷矿产量70万吨；压减煤炭产能75万吨，宜都、夷陵整体退出煤炭生产行业；淘汰落后产能企业24家，率先在全省淘汰20蒸吨以下燃煤锅炉。商品房库存面积和去化周期总体减少。降低企业综合成本100亿元以上。基础设施、基本公共服务等领域补短板步伐加快。

——人民群众获得感、幸福感和安全感持续增强。城镇新增就业10.6万人。城镇登记失业率2.06%。预计城乡居民人均可支配收入分别增长8%、8.5%。围绕“打通大动脉、疏通微循环”，城区完成畅通工程项目26个，打通“断头路”5条，新增公共停车位1472个。农房和居民自然灾害保险实现全覆盖。改造提升城区老旧小区128个，被纳入全国老旧小区改造城市试点。改造城区农贸市场44个。实现全国文明城市“三连冠”、全国社会治安综合治理长安杯“两连冠”，成为首批国家食品安全示范城市。

一年来，我们主要做了以下工作：

一是强化第一力度，抓实项目投资。坚持把项目投资作为稳增长的“定海神针”，强力推进招商引资“一号工程”，发展后劲持续增强。出台招商引资产业扶持政策，建立“周通报、月排名、季发言、半年拉练、年终结账”制度，完善考核评价办法，新开工亿元以上招商项目392个，增长3.2%。招商引资实际到位资金1642亿元，增长6.7%。实际利用外资3.7亿美元。争取国家和省预算内投资项目资金22.2亿元，落实三峡后续和对口支援资金148.8亿元。落地PPP项目22个、总投资169亿元。工业投资、民间投资占固定资产投资比重分别达到47%、67%。南玻智能触控显示器、世纪联合创新等项目建成投产，广汽传祺汽车生产基地、海航通航产业园、华强科技产业园、渝鄂直流背靠背工程等项目加快推进，页岩气勘探工作取得重大突破。三峡翻坝江北高速公路、伍家岗长江大桥、香溪长江大桥、白洋长江大桥等建设进展顺利。西陵二路、峡州大道二期、点军大道二期等加快建设，BRT夜明珠段、中南路延伸段、桔乡路等建成通车。建成一级公路57公里、二级公路280公里。硬化农村公路2442公里，实施生命安防工程4646公里。郑万高铁兴山段加快建设。白洋港一期开港营运。

二是加快结构调整，做强实体经济。坚持把结构调整作为促转型的有效途径，实施产业转型升级专项考评，下大力提升经济发展质效。出台化工产业专项整治意见和三年行动方案，规划建设宜都、枝江专业化工园区，沿江“一公里保护红线”正在形成。制定仿制药发展等扶持政策，新材料、生物医药、电子信息、先进装备制造业加快发展，产值占工业总产值比重达到40%。人福药业生产质量体系全面升级，原料及制剂通过美国和欧盟认证。净增高新技术企业57家、高新技术产品183个，实现高新技术产业增加值406.41亿元。发明专利授权量620件。成立全省第二家科技银行。实施“我选湖北·爱上宜昌”创新创业计划。我市被列为国家军民融合产业示范基地，跻身国家创新型特色城市行列。宜昌蜜桔、秭归脐橙成为全国百强农产品区域公用品牌，当阳、宜都、秭归成为全国首批有机肥替代化肥示范县，夷陵成为全国农业可持续发展示范区。

旅游环境综合整治成效明显。全年接待游客6618.23万人次，旅游总收入713.46亿元，分别增长17.02%、18.51%。《三峡临空经济区总体方案》获省政府批复。三峡机场年旅客吞吐量突破220万人次，增长45%。电子商务交易额1244亿元，增长38.2%。港口吞吐量8261万吨。现代物流总额增长14.1%。国家级三峡康养试验区创建工作加快推进。新引进金融机构7家。进入主板上市报会程序企业1家，上市企业再融资55.9亿元，企业债券融资175.7亿元。新增中国驰名商标5件、地理标志商标9件，安琪酵母荣获“中国质量奖提名奖”。出台统筹发展“飞地经济”政策，促进区域协调发展。宜都、夷陵、枝江、当阳、远安在全省县域经济分类考核中位居前列，秭归、兴山、长阳、五峰脱贫攻坚进程加快。

三是突出生态优先，加快绿色发展。坚持以“共抓大保护、不搞大开发”为导向，加大突出环境问题整治力度，坚定不移走绿色发展之路。编制《宜昌长江生态保护与绿色发展战略规划》，制定绿色发展指标体系，出台环境保护“一票否决”办法，协同制定《宜昌市黄柏河流域保护条例》，以压倒性力度推进长江宜昌段生态修复与治理。取缔非法码头151个，修复生态岸线41公里，关停沿江排污口26个。全面落实四级“河湖长”制，取缔江河湖库围栏围网养殖、珍珠养殖、投肥养殖。关闭禁养区规模养殖场134家。城区不达标水体治理加快推进，新建污水管网140公里，沙河、柏临河等河流水质明显好转。临江溪、点军污水处理厂提标建设按期完成，51个乡镇污水处理厂全部开工建设。出台建筑垃圾管理办法，强化工地扬尘管理，改装建筑渣土运输车417台，淘汰全部黄标车。市县两级设环保警察，创新“环保+公安”执法模式。认真实施《宜昌市城区重点绿地保护条例》，确定城区首批重点保护名录。建成城市绿道106.2公里。实施城区山体修复保护106公顷。绿化造林4.9万亩。划定永久基本农田397.3万亩。开工建设海绵城市7.2平方公里、城市地下综合管廊16.7公里。新增绿色建筑246.6万平方米。枝江成功创建国家园林城市。大老岭、崩尖子晋升为国家级自然保护区，青龙湖成为国家湿地公园，长阳清江国家地质公园获批复。兴山陈家湾、长阳郑家榜、当阳三里港入选全国美丽宜居村庄。

四是深化改革开放，激活内生动力。坚持把改革开放作为“关键一招”，着力推进重点领域、关键环节改革，积极解决制约经济社会发展的突出问题。湖北自贸区宜昌片区挂牌运行，入驻企业1561家。深化“放管服”改革，取消和调整市级行政审批等事项102项、中介服务事项36项，“四多合一”改革取得积极进展，“一窗办、一网办、帮你办”政务服务全面推行。出台市级政府投资项目管理、代建制管理、审计监督等办法，建立政府债务项目财政事前审批机制，政府债务管理全面加强。农村土地确权登记工作全面完成，“三权分置”改革、集体产权制度改革试点、国有林场改革有序开展。市属国有企业分类改革稳步推进，国有资本监管体制机制进一步理顺。公证、盐业、城市执法体制等改革顺利实施。深化医药卫生体制改革，城区公立医院全部取消药品加成，基层医疗卫生综合改革创新发展。城乡居民基本医疗保险制度完成整合，医保付费方式改革全面启动。机关事业单位养老保险制度改革、公务员职务与职级并行制度试点有序推进。积极融入“一带一路”战略，成功举办茶叶和柑橘国际采购商大会、国际茶旅融合论坛，宜汉欧货运班列加挂运行，新开通国际旅游包机航线2条。企业“走出去”步伐加快。三峡保税物流中心（B型）监管货值位居全省首位。外贸出口23.1亿美元，增长4%。

五是注重共建共享，增进民生福祉。坚持“用伟大心态做小事”，努力在发展中补齐民生短板、促进社会公平正义。财政民生支出达到392.3亿元，占一般公共预算支出比重为79.9%。大学生、退伍人员、去产能分流职工等重点群体就业帮扶力度加大。企业离退休人员养老金稳步增加，城乡低保、城乡特困人员供养标准两次提标。城镇职工、城乡居民医保政策范围内住院报销比例分别提高到75.6%、70.3%。棚户区改造开工2.31万套。改造农村危房1.8万户。巩固提升农村52万人饮水安全。完成281个小城镇（中心村）电网改造和126个村通动力电工程。

公共服务供给能力提升。新建、改扩建幼儿园25所，公办和普惠幼儿园覆盖率达到76%。义务教育均衡发展水平持续提升，普通高中形成优质化、多样化、特色化办学格局。市一中新校区、夷陵中学新校区、市青少年综合实践教育基地、市委党校新校区投入使用。职业教育产教融合步伐加快。三峡大学等在宜高校加快发展。三峡航空学院申报建设通过省级评估。特殊教育保障体系不断健全。社会主义核心价值观广泛弘扬，群众性精神文明建设成效显著。宜都获评全国文明城市，6个县市成为省级文明城市。国家公共文化体系示范区建设加快。成功举办“四节”。市一医院门诊急诊综合大楼、市二医院综合住院楼投入使用。乡镇卫生院、社区卫生服务中心等基层医疗机构建设提档升级。当阳被命名为国家卫生城市。宜昌健儿获得全运会3金3银3铜好成绩。成功举办市第五届运动会、宜昌国际马拉松赛。平安宜昌、法治宜昌建设取得新成效。“雪亮工程”深入推进。葛洲坝水利枢纽陆域实现封闭管理。“七五”普法全面开展。法律援助工作得到加强，办理法律援助案件3742件。

六是持续转变作风，提升治理能力。坚持把“为民、务实、清廉”作为价值追求，更大力度加强政府自身建设。严格落实全面从严治党主体责任，认真贯彻中央八项规定精神和省委、市委有关意见，推进“两学一做”学习教育常态化制度化，不断增强政府系统“四个意识”。认真落实省委巡视“回头看”整改任务。建立业务工作、党的建设、安全生产“三同步”责任区等制度。大力践行“一线工作法”，持续推进“转作风、抓落实”。加强行政监察和审计监督，严肃查处各类违纪违法案件。扎实开展行政执法专项治理，法治政府建设稳步推进。自觉接受市人大及其常委会法律监督和工作监督，主动接受市政协民主监督以及社会舆论监督，按期办复人大代表议案和建议367件、政协委员建议案和提案624件。与工会、共青团、妇联等群团组织联系更加紧密。

国防动员、人民防空、民兵预备役、双拥等工作进一步加强。援藏援疆、民族宗教、外事侨务、对台、信访、档案、保密、方志、粮食、供销、统计、科普、气象、消防、防震减灾、新闻出版、广播电视、政策咨询、社科、慈善、老龄、残疾人等工作取得新的成绩。

各位代表！事非经过不知难。过去的一年，改革发展任务之重超出预期，矛盾风险挑战之多超出预期，稳增长促转型考验之大超出预期，能取得这些成绩实属不易。这是省委省政府坚强领导、亲切关怀的结果，是市委统揽全局、科学决策的结果，是市人大市政协有效监督、大力支持的结果，更是全市人民团结一心、共同奋斗的结果。在此，我代表市人民政府，向全市广大干部群众，向各民主党派、工商联、无党派人士、各人民团体，向驻宜人民解放军、武警官兵，表示崇高的敬意！向关心支持我市改革发展的中央、省属在宜单位和海内外朋友，表示衷心的感谢！

各位代表！前进路上无坦途。总结一年来的工作，我们清醒地认识到，发展不平衡不充分的问题仍然突出，工作中还存在一些短板和弱项。主要是：化工产业转型升级任务艰巨，新兴产业体量小，服务业发展滞后，新旧动能转换还不够快；大项目、好项目较少，民间投资力度偏弱，部分企业生产经营困难，实体经济水平还不够高；医疗、教育、生态环保、安全生产、城区交通等领域民生难点解决得还不够好；少数部门和干部作风“宽、松、软、虚”，工作落实落地的力度还不够大。对这些问题，我们一定高度重视，不回避，不掩盖，采取坚决有力措施加以解决。

二、2018年工作重点

各位代表！凡是过去，皆为序章。今年是贯彻党的十九大精神的开局之年，是改革开放40周年，是决胜全面建成小康社会、实施“十三五”规划承上启下的关键一年。伴随着中国特色社会主义进入新时代，宜昌发展也踏上了新征程。历史只会眷顾坚定者、奋进者、搏击者，而不会等待犹豫者、懈怠者、畏难者。我们一定不忘初心、牢记使命，保持“行百里者半九十”的清醒，坚定“不破楼兰终不还”的决心，以永不懈怠的精神状态和一往无前的奋斗姿态，加快推进宜昌“绿色、转型、跨越、小康”进程，加快提升省域副中心城市辐射带动能力，创造无愧于新时代的新业绩！

根据中央经济工作会议和市委六届五次全会部署，我们将全面贯彻党的十九大精神，以习近平新时代中国特色社会主义思想为指导，坚持稳中求进工作总基调，践行新发展理念，紧扣社会主要矛盾，按照高质量发展要求，以供给侧结构性改革为主线，推动质量变革、效率变革、动力变革，加快构建现代化经济体系，打好三大攻坚战，加强和改善民生，促进经济社会持续健康发展，奋力建设社会主义现代化强市。

主要预期指标是：生产总值增长7%以上，规模工业增加值增长7%以上，固定资产投资增长11%，社会消费品零售总额增长11%，外贸出口总额增长8.2%，城乡居民收入增长高于经济增速。

约束性指标是：万元生产总值能耗下降3.7%、地耗下降5.32%、水耗下降7%；空气质量优良天数比例达到73%，全面完成省政府下达的污染减排和环境保护任务；城镇登记失业率控制在4%以内。

做好今年的政府工作，必须正确处理“四个关系”：一是速度与质效的关系。既要确保经济运行在合理区间，为转型升级创造有效空间和条件，更要坚持质量第一、效益优先，为经济发展增添新动力，实现更高质量、更有效率、更加公平、更可持续的发展。今年主动调低预期指标，考虑了转型需要，突出了高质量导向，体现了稳中求进。二是发展与生态的关系。良好的生态是宝贵的发展资源。必须树牢和践行“绿水青山就是金山银山”的理念，实行最严格的生态环境保护制度，坚定走生产发展、生活富裕、生态良好的文明发展之路，实现人与自然和谐共生。今年将约束性指标与预期指标并列，就是树立更加鲜明地绿色发展导向。三是发展与民生的关系。增进民生福祉是发展的根本目的。必须牢固树立以人民为中心的发展思想，多谋民生之利、多解民生之忧，坚持“用伟大心态做小事”，让改革发展成果更多更公平惠及全体人民。四是当前与长远的关系。发展不能搞短期行为，更不能急功近利。必须以“功成不必在我”的境界，夯实发展基础、补齐发展短板、打牢发展根基，扎实做好打基础、利长远的事。

今年，我们将围绕推动高质量发展，做好七项重点工作：

（一）致力推动经济稳健增长。“稳”是为了更好地“进”。我们必须抓住经济运行中的突出问题，对症下药、精准发力，推动经济行稳致远。

发挥有效投资关键作用。优化招商引资和重点产业项目建设专项考核办法，坚持用第一力度抓项目抓招商。完善重大项目推进机制，对省市重点项目实行“定时、定项、定向”调度，促进项目尽快落地开工、竣工投产。以产业项目特别是制造业项目为发力点，加快推进广汽传祺汽车生产基地、三宁化工年产60万吨乙二醇等新建项目，东阳光年产25吨冬虫夏草、江升锂离子动力电池隔膜材料等一批续建项目。突出产业链招商，深化以商招商、委托招商、园区招商，重点引进一批龙头型、配套型项目，力争招商引资到位资金1600亿元，新开工亿元以上招商项目400个。深化与中省企业战略合作，扩大与三峡对口支援单位经济协作，推动一批项目尽快落地见效。

精准服务实体经济发展。着力构建“亲”“清”新型政商关系，常态化开展“联企业、解难题”活动。坚持“一企一策”，引导骨干企业聚焦主业、转型升级，着力培育一批行业龙头和细分市场冠军。 支持中省在宜企业做强做优做大。支持企业增品种、提品质、创品牌。深入推进产融合作试点，统筹用好生产调度资金、产业基金，强化财政金融联动，增强金融服务实体经济能力。力争直接融资200亿元，新增上市公司1家。完善扶持民营经济发展政策，落实好减税降费各项举措，推动民营经济主体增加、投资增长、质效增强。加快“小进规”“小进限”步伐，力争规上、限上企业分别新增100家。

企业是经济的基本细胞，企业家是宝贵资源。我们将着力营造企业家健康成长环境，弘扬优秀企业家精神，更好发挥企业家作用，让企业家在振兴实体经济中创造新业绩、铸就新辉煌！

着力防范化解重大风险。引导企业强化风险意识，合理控制资产负债率；更好发挥债权人委员会作用，引导金融机构帮助困难企业渡过难关。严格规范政府举债融资行为，加快市县属国有投融资公司市场化转型，规范PPP项目操作，坚决遏制隐性政府债务风险增量。加强不良资产、债券违约、影子银行、企业互保等领域金融风险防控，坚决打击违法违规金融活动，坚决守住不发生系统性区域性风险的底线。

（二）致力加快新旧动能转换。动能迭代更替是转型升级的内生动力。我们必须坚持先进制造业和现代服务业“双轮驱动”、传统产业升级和新经济培育“双措并举”，让新动能尽快成为主导力量。

大力发展先进制造业。深入实施《中国制造2025宜昌行动纲要》，围绕新材料、生物医药、高端装备、新一代信息技术、节能环保与新能源等五大重点领域，培育壮大战略性新兴产业。支持企业开展仿制药一致性评价，努力打造国内一流的仿制药生产基地。出台扶持政策，着力培育汽车零部件产业。推动船舶与海洋工程装备、民用航空航天等领域的产业项目尽快落地，加快建设国家军民融合产业示范基地。积极推进页岩气、风能、太阳能等新能源产业化，努力打造“清洁能源之都”。深入实施智能制造专项行动，引导更多企业实施智能化改造。扩大“两化”融合对标试点范围，确保新增国家级贯标认定企业2家。

推动传统产业优化升级。设立1亿元产业转型升级专项资金，加快实施安琪健康食品数字化车间新模式应用、三峡制药猇亭硫酸新霉素基地、三峡烟厂易地技改搬迁等67个重点技改项目，确保技改投资高于工业固定资产投资增长水平。强力推进化工产业转型升级，高标准建设宜都、枝江专业化工园区，引导符合标准的化工企业向园区集中，化工产品向高效环保肥料、精细专用化学品、高性能功能材料转型。大力发展绿色健康食品产业。加快发展装配式建筑产业。深入创建全国质量强市示范城市。力争新增地理标志商标2件以上、湖北名牌产品20个以上。

突破性发展现代服务业。设立3000万元服务业发展引导资金，实施三峡大学科技文化产业园、枝江农旅电商物流园等一批重点项目，促进服务业提质增量，力争服务业增加值增长12%以上。加快发展生产性服务业。以园区物流、专业市场物流、产业集群物流、城乡配送物流为重点，引进培育一批物流骨干企业，力争A级企业达到70家。支持金融机构做强做优。加快发展商务会展、研发设计、检验检测认证等产业。提升生活性服务业品质。深入实施千亿旅游产业三年行动计划，力争旅游总收入增长20%。加快整合“两坝一峡”旅游资源，全力攻坚平湖半岛征迁，积极创建三峡国家级旅游度假区。支持三峡大坝景区转型增效，支持三峡大瀑布创建5A级景区。启动长阳火烧坪、远安太平顶、点军白云山等度假小镇建设。支持枝江、兴山、长阳新建通用机场。继续推进12条旅游风景道建设。积极发展文化产业。打造具有浓郁地方特色的精品文化旅游大戏。加快推进重点商圈和特色商业街区建设。推动城区农贸市场改造升级。办好第28届中国厨师节。培育综合性服务业发展亮点。大力发展电商产业，力争交易额突破1500亿元。加快发展康养产业，推进亚行贷款养老综合服务、山城水都健康城、卓尔国际旅游小镇、榛子高山旅游康养小镇等项目建设。

全面激发“双创”活力。深入实施创新驱动发展战略，加快建设区域性创新中心和国家创新型城市。持续推进“科技型中小企业创新成长工程”“高新技术企业倍增工程”，确保新增高新技术企业40家以上、高新技术产品130个以上，实现高新技术产业增加值增长8%。大力发展数字经济，加快建设数据存储容灾备份中心。新增市级研发平台8家以上，积极创建国家级、省级研发平台。加快孵化培育创新型小微企业，新增在孵企业100家以上。加大中小企业创新支持力度，促进科技成果在宜转化，完成转移扩散和转化应用项目100项以上。支持宜昌高新区、西陵区创建“全国双创示范基地”。强化知识产权创造、保护、运用，力争发明专利授权量突破550件。落实“人才新政十八条”，努力让各类人才的创造活力竞相迸发、聪明才智充分涌流。

（三）致力加强生态文明建设。绿色是宜昌的基本底色，生态是宜昌最大的优势。我们必须坚持节约优先、保护优先、自然恢复为主的方针，建设好“美丽宜昌”，将“绿水青山”的生态优势转化为“金山银山”的发展优势。

着力解决突出环境问题。环境污染是民生之患、民心之痛，必须重拳出击，铁腕整治。重拳治理大气污染。统筹推进“减煤、治源、控车、抑尘、禁烧”等重点工作，确保主要环境空气污染物浓度持续下降，打赢蓝天保卫战。重拳治理水污染。依法推进沿江82家化工企业关停、搬迁。确保工业园区污水集中处理设施全部投入运营、51个乡镇污水处理设施全部建成。按照“八先八后”原则，完成城区11条不达标水体整治。加强沿江排污口综合治理。坚决取缔非法码头，推动建设综合性码头，促进岸线资源高效集约利用。实行最严格的饮用水源地保护制度。开展总磷专项治理。重拳治理土壤污染。启动工矿企业土壤污染防治工作。完成农用地土壤环境质量详查。加强农业面源污染、畜禽养殖污染综合整治。加快推进第二次污染源普查。全面推进领导干部自然资源资产离任审计。严格跟踪问效，全面、高质量完成中央环保督察整改任务。

强化生态保护与修复。坚决管住斧头、守住山头、护好源头，严守空间底线、发展底线、民生底线和城镇开发边界线。深入推进长江宜昌段生态修复与治理。加快建设沙河片区综合开发、柏临河湿地公园等一批海绵城市试点项目。加快推进磨基山公园、求雨台公园、松林公园等项目建设。完成城区山体修复保护300公顷。坚持“四季挖窝、三季植绿”，复绿长江岸线1600亩，精准灭荒8000亩。确保通过国家森林城市复查。完善“河湖长”制，全面推进河道生态治理与修复。抓好黄柏河东支流域生态补偿试点。治理水土流失面积260平方公里。加大矿山地质环境恢复治理力度。

推进绿色低碳循环发展。实施能源消费、水资源消耗、建设用地等总量和强度控制，推进磷矿开采总量与矿权数量双控行动。确保国家节水型城市创建通过验收。探索用能权、排污权有偿使用和交易。推进碳排放企业纳入全国统一市场管理。完善控制污染物排放许可制度。推进磷石膏等大宗固体废弃物综合利用。支持工业园区循环化改造。全面推行城市生活垃圾分类工作。抓好“岸电工程”“气化乡镇”等试点示范工程。大力推广绿色建筑、节能建筑、可再生建筑。鼓励在环境优先区域实施清洁能源汽车替代工程。开展创建节约型机关、绿色家庭、绿色学校、绿色社区和绿色出行等行动。

绿色生态是最好的民生、最大的福利。我们一定全力回应人民群众盼环保、求生态的热切期待，让一半山水一半城，山在城中、城在山中的宜人之城天更蓝、山更绿、水更清，让世界水电旅游名城更加美丽！

（四）致力全面深化改革开放。改革开放是决定转型成败的根本之策。我们必须坚持改革不停顿、开放不止步，勇涉“深水区”、敢啃“硬骨头”，以实际行动迎接改革开放40周年。

深化供给侧结构性改革。坚持用市场机制、法治手段、环保安全标准推进“去产能”，依法依规处置“僵尸企业”，全部关停现有煤矿，如期退出煤炭生产行业。坚持“房子是用来住的、不是用来炒的”定位，完善促进房地产市场平稳健康发展的长效机制。积极培育发展住房租赁市场特别是长期租赁，支持专业化、机构化住房租赁企业发展。实施新三年棚改计划，今年开工1.48万套。综合运用股权融资、市场化债转股等方式，积极稳妥降低企业杠杆率。持续打好降低制度性交易成本、物流成本“组合拳”，继续清理涉企收费，推动企业“眼睛向内”降本增效。实施补短板“十大重点工程”。

加快推进关键领域改革。继续下放一批行政审批事项，加强基层承接能力建设。全面实行市场准入负面清单制度，稳步推进“证照分离”。深化“四多合一”，探索“多规合一”。完善“互联网＋政务服务”，努力让“网上办”成为常态、“网下办”变成例外。推进“双随机一公开”监管全覆盖。加强中介服务管理平台在线应用。加快交通、农业、城管等领域综合执法改革。加强社会信用体系建设。规范国企法人治理结构，强化国企风险管控，促进国有资产保值增值。完成事业单位、国有企业公务用车制度改革。深化税收征管体制改革。支持中省在宜企业“三供一业”及社会职能分离移交。积极推进自然资源资产负债表、资源出让与环境治理挂钩、环保执法垂直管理等生态文明制度改革。深入推进农村土地“三权分置”、集体产权制度等改革。启动新一轮政府机构改革。

今年，我们将以更大决心多推有利于增添经济发展动力的改革，多推有利于促进社会公平正义的改革，多推有利于增强人民群众获得感的改革，多推有利于调动广大干部群众积极性的改革，努力扩大改革的受益面！

提升开放合作水平。抓住国家扩大自贸区改革自主权的重大机遇，以制度创新为着力点，在政府职能转变、投资环境、法治建设等领域寻求更大突破，推动高新产业、现代服务业在自贸区集聚，加快把宜昌自贸片区打造成三峡区域对接“一带一路”战略的重要节点。有序实施汽车平行进口、国际快件监管中心等功能性项目。加快推进综合保税区申报建设，更好发挥三峡保税物流中心平台功能。全面推进国际贸易“单一窗口”应用。加快宜昌港对外开放，促进宜昌中欧班列、水运班轮常态化运营。推动三峡机场扩大开放，力争出入境旅客突破5万人次。引导企业“走出去”开展投资和经贸合作，助力企业开拓国际市场。深化茶叶、柑橘“国际采购商宜昌行”活动。加快培育电子信息、生物医药、农产品等外贸竞争新优势。

（五）致力实施乡村振兴战略。“三农”问题是关系国计民生的根本性问题。我们必须按照产业兴旺、生态宜居、乡风文明、治理有效、生活富裕的总要求，加快推进农业农村现代化。

坚决打好脱贫攻坚战。严格落实脱贫攻坚责任制，下足“绣花”功夫，力争兴山和秭归摘帽、55个贫困村出列、104671人脱贫，13849人易地扶贫搬迁。深入开展“双建双包双带双促”帮扶行动，健全大扶贫工作格局。加大产业扶贫力度，强化就业扶贫、健康扶贫、教育扶贫、社保扶贫，拓展光伏扶贫、金融扶贫、电商扶贫等新模式，增强可持续发展能力。向深度贫困地区聚焦发力，对五峰深度贫困县、40个深度贫困村进行重点帮扶。注重扶贫与扶志、扶智相结合，激发贫困人口内生脱贫动力。坚决纠正扶贫领域不正之风，严厉惩治扶贫领域腐败问题。

加快发展现代特色农业。深入推进农业供给侧结构性改革，着力打造绿色农业、品牌农业、开放农业、科技农业，推进农业生产由增产向提质转变。加快调整柑橘熟期品种结构，大力推广无性系茶叶良种，稳步扩大精细蔬菜比重，积极发展特色水产健康养殖和畜禽绿色养殖。加快林业特色产业、中药材产业发展。强化农产品公用品牌建设，着力打造“宜昌蜜桔”“宜昌宜红”“宜昌猕猴桃”城市新名片。积极开展农产品质量安全县市创建工作。大力发展农产品加工业和休闲农业，促进农村一二三产业融合发展。支持建设一批高品质、上档次的田园综合体。提升农村电商发展水平。开展农业关键环节科技攻关，加大新品种、新技术、新设施的推广运用。

推进城乡融合发展。健全城乡融合发展体制机制，清除阻碍要素下乡各种障碍，实施市民下乡、能人回乡、企业兴乡“三乡工程”。支持安福寺绿色食品小镇、高坝洲柑甜小镇建设。加强传统村落、特色村寨保护。扎实推进“厕所革命”。大力开展“消危减土”行动，改造危房1.2万户。整村推进55个建档立卡贫困村饮水安全巩固提升任务。加快抗旱骨干水源工程建设，新增高效节水灌溉面积8万亩。加快推进清江、沮漳河、香溪河、渔洋河等防洪治理项目。硬化农村公路1500公里，实施生命安防工程3500公里。建设高标准基本农田48万亩。

（六）致力推进区域协调发展。区域协调发展是全面建成小康社会的内在要求。我们必须注重发展的整体性、协调性，推动城乡、区域之间的发展向更加均衡、更高层次迈进。

提升城市功能品质。加快推进伍家岗长江大桥建设，确保西陵二路10月份全线建成通车，夷桥路快速化改造工程、点军大道二期、花溪路伍家岗大桥至柏临河段年内建成通车，加快峡州大道二期、江城大道二期、夷陵区“三路一匝”等建设，实现快速路网内外环基本闭合、中环形成骨架。完成点军片区16.7公里地下综合管廊主体工程建设。确保奥体中心、博物馆基本完工，妇女儿童活动中心、体育馆投入使用。持续推进五龙、奥体中心、唐家湾、东站、共联等片区深度开发，抓好高铁北站片区规划。持续实施城区畅通工程，积极创建“国家公交都市”，完成智慧停车系统建设，新增公共停车位2000个以上。抓好全国老旧小区改造试点，完成改造试点10个以上。不断提升城市精细化管理水平。

打造综合交通枢纽。力争开工建设呼南高铁宜昌段，开工建设郑万高铁连接线、远当铁路，加快“县县通铁路”进程。积极推进城市轨道交通建设。加快推进三峡机场二期改扩建项目。力争开工建设宜来高速、宜张高速鄂湘界段，加快建设白洋长江公路大桥、三峡翻坝江北高速，力争神农架至五峰高速、当阳经枝江至松滋高速工程可行性研究报告获批，力争“县县通达纵横2条高速”纳入省“十三五”中期调整计划。开工建设宜都红花套至长阳偏岩一级公路、三斗坪旅游客运港改建、宜昌东站物流中心等30个重点项目，加快推进香溪长江公路大桥、白洋港二期等50个在建重点项目。确保域内城际公交全覆盖。

构建多极发展格局。支持宜昌高新区发挥叠加优势，推进专业园区开发建设，加快打造全国一流开发区。支持宜昌新区提升建设品质，打造生态宜居城市。支持三峡旅游新区加快打造旅游产业聚集区、品牌形象展示区。支持三峡临空经济区打造全国知名的临空产业示范基地、港产城融合发展的绿色智慧航空新城。支持三峡枢纽港打造长江三峡航运物流中心。支持西陵、伍家岗、点军、猇亭发展各具特色的都市经济。支持宜都、夷陵、枝江、当阳、远安提升实力、争先进位。支持长阳、秭归、五峰、兴山补齐短板、奋力赶超。鼓励缺乏工业发展要素支撑的地方，到宜昌高新区、东部县市专业园区发展“飞地经济”。通过支持各地突出特色、错位发展，尽快形成优势互补、协调并进的区域发展新格局。

（七）致力提高民生保障水平。民生是最大的政治。我们必须始终把人民利益摆在至高无上的地位，从人民最关心最直接最现实的利益问题入手，尽力而为、量力而行，把实事办实、好事办好，让人民的获得感、幸福感、安全感一年更比一年强。

不断提升就业质量。坚持就业优先战略和积极就业政策，实现更高质量、更加充分就业。深化职业技能培训，解决就业结构性问题。最大限度扶持创业，以创业带动就业。发挥各项就业新政的促进作用，统筹抓好城镇登记失业人员、高校毕业生、农村劳动力、城乡就业困难人员等人群就业创业，新增城镇就业7.6万人。深化和谐企业创建，构建和谐劳动关系。

加强社会保障体系建设。全面实施全民参保计划。完成机关事业单位养老保险制度改革任务。继续推进跨省异地就医直接结算，深化复合式医保付费方式改革。全面推进建设领域参加工伤保险。健全社会救助体系，加强农村“三留守”人员关爱服务和困境儿童保障，努力让弱势群体困有所帮、弱有所扶。加快构建以居家为基础、社区为依托、机构为补充、医养相结合、城乡全覆盖的养老服务体系。扩大高龄老人意外伤害保险覆盖面。

完善公共文化服务体系。大力实施文明创建提质惠民三年行动计划，健全完善长效机制，巩固提升创建成果。支持枝江争创全国文明城市。确保国家公共文化服务体系示范区创建通过验收。推出一批文艺精品力作，着力培养德艺双馨的名家大师和高水平创作人才。推动万里古茶道申遗工作。抓好国家文化消费试点，扩大文化消费。广泛开展全民健身活动。办好屈原故里端午文化节、全国举重锦标赛等节赛活动。加强互联网内容建设，营造清朗的网络空间。 办好人民满意的教育。坚持教育优先发展，落实立德树人根本任务，发展素质教育，推进教育公平，着力解决“择校热”“大班额”等突出问题，努力让每个孩子都能享有公平而有质量的教育。加强和规范幼儿园管理，新建、改扩建幼儿园20所。进一步优化中小学布局，推动义务教育优质均衡发展。深化高中阶段教育结构性改革。完善职业教育和培训体系，推进职业教育现代学徒制改革，深化产教融合、校企合作。启动新一轮特殊教育提升计划。支持三峡大学“双一流”建设和三峡职院创建全国优质专科高职学院，力争三峡航空学院申办成功。支持和规范民办教育发展。加强师德师风建设，培养高素质教师队伍，倡导全社会尊师重教。

着力打造“健康宜昌”。抓好健康城市国家试点，努力为人民提供全方位全周期健康服务。坚持医疗、医保、医药联动，不断深化医药卫生体制改革，力争在分级诊疗、医联体、全民医保、药品供应保障、现代医院管理等方面取得新突破。加快推进市中心医院综合楼等项目建设，着力打造区域性医疗卫生中心。鼓励社会资金进入养老、医疗等领域。传承发展中医药事业。做好“全面两孩”配套服务。深化国家食品安全示范城市创建成果，推动食品安全城市创建向县市延伸。落实粮食安全行政首长负责制，加强放心粮油体系建设，创建一批“放心肉菜示范超市”。

加强和创新社会治理。推进社会治理共建共治共享，不断提高社会治理社会化、法治化、智能化、专业化水平。继续推进城乡网格化服务管理。加强矛盾纠纷调处化解，做好重大项目社会稳定风险评估，及时预防化解各类群体性问题。树牢安全发展理念，弘扬生命至上、安全第一的思想，铁心、铁面、铁腕、铁纪、铁痕抓好安全生产，深入开展“打非治违”专项行动，确保实现“一无一降”目标。健全公共安全体系，提升防灾减灾救灾能力。深化平安宜昌建设，完成全国“雪亮工程”示范城市建设任务，实现全域覆盖、全网共享、全时可用、全程可控目标，依法严厉打击违法犯罪活动。推进公共法律服务全覆盖。

支持驻宜部队建设改革，加强国防教育和国防后备力量建设，巩固军政军民团结的良好局面。继续做好援藏援疆、民族宗教、外事侨务、对台、人防、供销、档案、保密、方志、气象、消防、科普、社科、慈善、残疾人等工作。

三、全力打造人民满意政府

各位代表！民之所望，政之所向。我们必须始终坚持以人民为中心的发展思想，把人民对美好生活的向往作为奋斗目标，把执政为民的价值追求贯穿到政府工作各领域全过程，不断提升政府治理能力和水平。

必须牢记使命、忠诚为民。提高政府工作的政治站位。坚持用习近平新时代中国特色社会主义思想武装头脑，强化“四个意识”，坚定“四个自信”，将忠诚为民融入血液、铸入灵魂，坚定不移做“不忘初心、牢记使命”的践行者。坚决贯彻落实党中央国务院大政方针、省委省政府决策部署，始终把政府工作置于市委领导之下，确保政令畅通、令行禁止。坚持深入基层、根植大众、服务人民，履行好人民公仆职责。坚守节约裕民的正道，牢固树立过“紧日子”思想，将有限的财力更多用在民生保障和改善上，让民生幸福的底色更加厚重，让共享发展的主题更加温暖！

必须敢于担当、奋发进取。有多大担当才能干多大事业。在改革发展稳定各项工作中，始终冲在前面、干在实处，积极应对重大挑战、抵御重大风险、克服重大阻力、解决重大矛盾，争做“主攻手”、不当“二传手”。强化“五种思维”，锤炼“八大本领”，知难而进、迎难而上、破难而行，以滚石上山的精神，努力把看似干不成、过去干不成、别人干不成的事情，想方设法干成干好、干出实效！

必须务实重行、提升效能。务实笃行才能精彩出彩。大兴学习、调研之风，自觉践行“一线工作法”，切实把抓改革、谋转型、促发展的任务落实到具体项目上，把满足人民对美好生活向往的承诺落实到具体行动上。营造干事创业的良好环境，撸起袖子、扑下身子、心无旁骛抓落实，争做“工匠+猛将”型干部。着力提高政府执行力，切实做到“文不过夜、事不隔天”。优化政务工作流程，推动政府工作进一步提速、提质、提效，尽力让群众和企业“少跑腿、好办事、不添堵”！

必须依法行政、正风肃纪。秉公用权才能取信于民。坚持法定职责必须为、法无授权不可为，严格规范公正文明执法，全面推进法治政府建设。严格执行人大及其常委会决议决定，提高人大代表议案、建议和政协委员建议案、提案办理质量，自觉接受人大及其常委会的法律监督和工作监督以及政协民主监督，认真听取民主党派、工商联、无党派人士和各人民团体意见，高度重视社会监督和舆论监督。健全民主科学决策机制，使政府的每一项决策都遵从法律法规、体现社情民意、彰显公平正义。严格落实全面从严治党主体责任，严格落实中央八项规定精神及省委市委实施办法，严格执行市委转作风抓落实“六个坚持、六个严禁”要求，驰而不息整治“四风”。坚决纠正损害群众利益的不正之风，坚决查处腐败案件，做到惩治力度决不减弱、零容忍态度决不改变、廉洁从政要求决不放松！

各位代表！身处伟大时代，我们无尚荣光；肩负伟大使命，我们责重千钧。让我们更加紧密团结在以习近平同志为核心的党中央周围，在省委省政府和市委坚强领导下，振奋精神、锐意进取，埋头苦干、攻坚克难，汇聚绿色发展、转型跨越、决胜小康的磅礴力量，为建设社会主义现代化强市而努力奋斗！
